# Behavioural and morphological evidence for the involvement of glial cell activation in delta opioid receptor function: implications for the development of opioid tolerance

**DOI:** 10.1186/1744-8069-3-7

**Published:** 2007-03-12

**Authors:** Sarah V Holdridge, Stacey A Armstrong, Anna MW Taylor, Catherine M Cahill

**Affiliations:** 1 Department of Pharmacology & Toxicology, Queen's University, Kingston, Ontario, K7L 3N6, Canada; 2 Department of Anesthesiology, Kingston General Hospital, Queen's University, Kingston, Ontario, K7L 2V7, Canada

## Abstract

Previous studies have demonstrated that prolonged morphine treatment *in vivo *induces the translocation of delta opioid receptors (δORs) from intracellular compartments to neuronal plasma membranes and this trafficking event is correlated with an increased functional competence of the receptor. The mechanism underlying this phenomenon is unknown; however chronic morphine treatment has been shown to involve the activation and hypertrophy of spinal glial cells. In the present study we have examined whether activated glia may be associated with the enhanced δOR-mediated antinociception observed following prolonged morphine treatment. Accordingly, animals were treated with morphine with or without concomitant administration of propentofylline, an inhibitor of glial activation that was previously shown to block the development of morphine antinociceptive tolerance. The morphine regimen previously demonstrated to initiate δOR trafficking induced the activation of both astrocytes and microglia in the dorsal spinal cord as indicated by a significant increase in cell volume and cell surface area. Consistent with previous data, morphine-treated rats displayed a significant augmentation in δOR-mediated antinociception. Concomitant spinal administration of propentofylline with morphine significantly attenuated the spinal immune response as well as the morphine-induced enhancement of δOR-mediated effects. These results complement previous reports that glial activation contributes to a state of opioid analgesic tolerance, and also suggest that neuro-glial communication is likely responsible in part for the altered functional competence in δOR-mediated effects following morphine treatment.

## Background

The opioid system, comprised of multiple highly homologous receptor families and their endogenous opioid peptide ligands, is fundamental to the modulation of the sensory and affective aspects of pain [1]. Three classes of opioid receptors (ORs) have been identified through molecular and pharmacological techniques, namely the mu (μ), delta (δ), and kappa (κ) ORs [reviewed by 2, 3]. Morphine, a classical μOR agonist with remarkable analgesic efficacy, is the current gold standard in the clinical treatment of moderate to severe pain; however, its use in the management of chronic pain may be restricted by the development of analgesic tolerance and the unwanted side effects associated with dose escalation. As such, understanding the mechanisms underlying opioid tolerance has become the primary focus of an extensive research effort with the aim of uncovering novel therapeutic strategies to treat persistent, unremitting pain.

A growing body of evidence identifies the δOR as an instrumental player in the development of morphine-induced analgesic tolerance [reviewed by 4]. Thus, concomitant administration of δOR antagonists with morphine [5-9] or antisense oligodeoxynucleotide treatment directed against the δOR [10] partially blocked the development of tolerance to morphine antinociceptive effects. In agreement with this data, δOR null mutant mice had a lower propensity to develop antinociceptive tolerance to morphine compared to their wild type littermates [11,12]. The mechanism by which δOR modulates μOR analgesic tolerance is not presently known, however, complex interactions between μ and δORs are likely to be relevant in eliciting various opioid-induced physiological responses. For example, direct coupling of μ-δORs in the form of hetero-oligomers has been demonstrated in both expression systems and spinal cord tissue [13], which was proposed to underlie the antinociceptive synergy between μ and δOR agonists. We, and others, have also demonstrated that chronic activation of the μOR induces a translocation of δORs from intracellular compartments to neuronal plasma membranes and this phenomenon is correlated with an increase in δOR functional competence [14-18]. Taken together, the activation and translocation of δORs may represent an important intermediary step in the development of morphine tolerance; however the mechanism underlying this trafficking remains unknown.

Several studies suggest an intimate and interactive relationship between opioids and glial cells. Once regarded as mere supports cells for CNS neurons, glial cells are now recognized as performing vital and complex functions in response to physiological stressors. Indeed, spinal glial activation has been observed in a number of pathological states including Alzheimer's [19,20] and Parkinson's [21] diseases, HIV-associated dementia [22-24], as well as several persistent pain syndromes [25-30]. Moreover, spinal glial cell activation has been linked to the development of opioid tolerance. Chronic morphine treatment was reported to activate microglial [31] and astrocytic [31,32] cells and to increase pro-inflammatory cytokine levels [31] in the lumbar spinal cords of tolerant rats. Accordingly, co-administration of a glial modulatory agent with morphine attenuated the spinal immune response and inhibited the loss of morphine analgesic potency [31,32], suggesting that spinal glia may contribute to mechanisms responsible for opioid tolerance.

In the current study, we aimed to investigate the functional relationship between δORs and glial cells following prolonged chronic morphine administration. We employed immunohistochemical techniques as well as a behavioural nociceptive paradigm to assess whether prolonged morphine treatment is associated with the activation of spinal glial, and if indeed so, whether this spinal immune response is requisite for the observed enhancement in δOR-mediated antinociception.

## Results

### Prolonged morphine treatment induces spinal astrocytic and microglial hypertrophy

**Figure 1:**
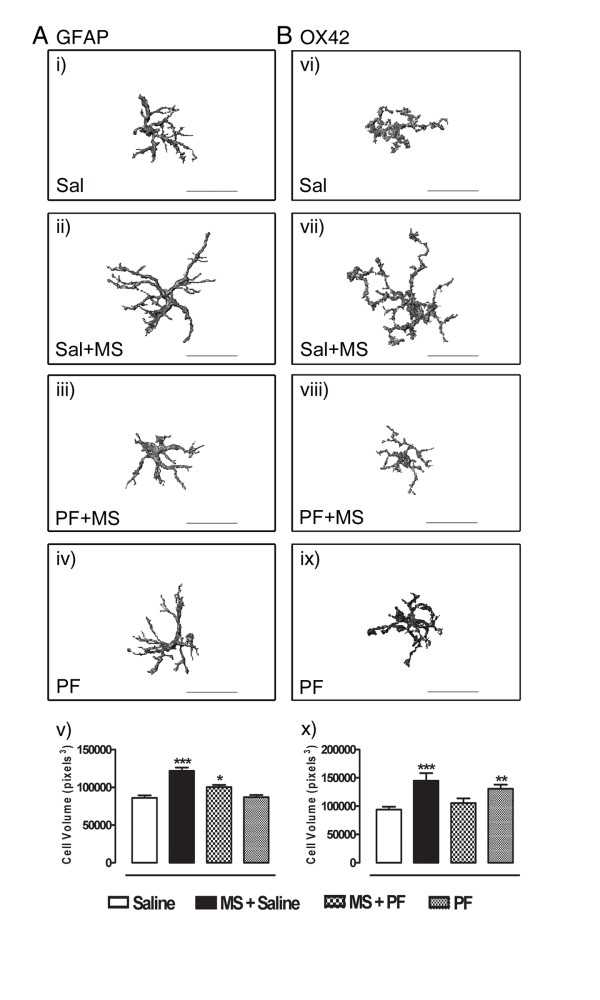
Detection of GFAP (panel A) and OX42 (panel B) in the dorsal lumbar spinal cords of rats treated, or not, with morphine was performed by fluorescent immunohistochemistry and photomicrographs were acquired by confocal microcopy. Displayed are representative three dimensional images of immunoreactive cells from rats receiving intrathecal saline (i, vi), morphine and intrathecal saline (ii, vii), morphine and intrathecal propentofylline (iii, viii), or intrathecal propentofylline alone (iv, ix). Morphine treatment produced a significant increase in both astrocytic and microglial cell volumes as compared with control. This hypertrophy was attenuated by coadministration of morphine with propentofylline. While propentofylline alone had no effect on GFAP-immunostaining, it significantly enhanced OX42-immunoreactive cell size. Data represent means ± s.e.m. for n = 12–20 cells per rat from n = 3 rats per group. Statistical analyses were performed by a one-way ANOVA followed by Tukey's *post-hoc *multiple comparison test. The *asterisk *denotes significant difference from saline-treated rats. * = p < 0.05, ** = p < 0.01, *** = p < 0.001. MS: morphine sulfate; PF: propentofylline; Sal: saline. Scale bar, 30 μm.

The effects of morphine treatment (5–15 mg/kg every 12 h; s.c.) on spinal astrocytes and microglia were assessed by fluorescent immunohistochemistry for confocal microscopy. Lumbar spinal cord segments from rats treated, or not, with morphine and/or the glial modulating drug, propentofylline (10 μg/30 μl; intrathecally [i.t.]), were processed for fluorescent detection of both GFAP and OX42, markers of astrocytes and microglia, respectively. Morphine treatment produced a significant increase in astrocytic (F_3,189 _= 23.79, p = 0.0015; Figure 1) and microglial (F_3,101 _= 8.403, p < 0.0001; Figure 1) cell volume as compared with saline-treated rats, indicating glial cell hypertrophy. Similarly, the surface areas of both astrocytic and microglial cells were significantly greater following morphine treatment as compared with saline-treated rats (F_3,189 _= 18.70, p = < 0.0001 for GFAP; F_3,101 _= 10.32, p = < 0.0001 for OX42; Table 1). Chronic intrathecal propentofylline administration effectively attenuated this morphine-induced hypertrophy, inhibiting both the increases in cell volume and in cell surface area. Propentofylline administration alone had no effect on astrocytic cell size as it produced no change in GFAP-immunoreactivity compared with controls. Interestingly, propentofylline alone significantly increased the cell volumes and surface areas of OX42-immunoreactive cells, indicating an effect on microglia which was independent of morphine treatment.

**Table 1:** Cell Surface Area (pixels^2^)

	**Saline**	**Saline + MS**	**PF + MS**	**PF**
**GFAP**	41843.41 ± 1603.904 ***	57440.11 ± 1960.131	48338.16 ± 1667.994 **	43235.54 ± 1369.27 ***
**OX42**	50418.92 ± 2441.907 ***	79908.27 ± 6287.605	55693.48 ± 4137.721 **	67083.38 ± 3756.654

Total cell surface area in pixels^2 ^was calculated from the three dimensional reconstructed images of GFAP- and OX42-immunopositive cells within the dorsal spinal cords of rats treated with saline, saline and morphine, propentofylline and morphine, or propentofylline alone. Data represent means ± s.e.m. for n = 12–20 cells per rat from n = 3 rats per experimental group. Statistical analyses were performed by one-way ANOVA followed by Tukey's *post-hoc *multiple comparison test. The *asterisk *denotes significant difference from rats treated with saline and morphine. ** = p < 0.01, *** = p < 0.001. MS: morphine sulfate; PF: propentofylline.

### Morphine-induced enhancement in δOR-mediated antinociception is attenuated by propentofylline

**Figure 2:**
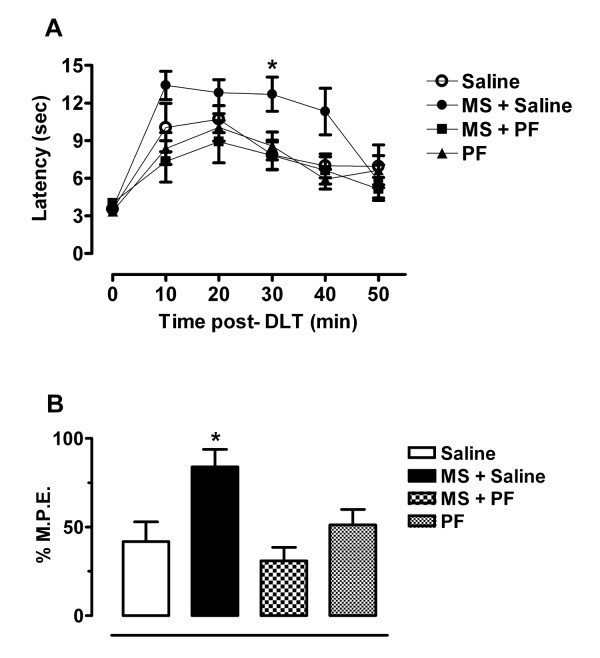
The antinociceptive effects of DLT (10 μg, i.t.) were assessed in the tail flick acute thermal pain test. Rats chronically treated with morphine exhibited enhanced δOR-mediated analgesia as compared with controls and this enhancement was blocked by chronic co-administration of morphine with the glial modulatory agent, propentofylline. All testing was performed 12 h following the final morphine injection. A) The latencies to respond with a brisk tail flick were measured prior to and at 10 minute intervals following DLT administration for 50 minutes. Three pre-drug latencies were averaged to obtain a baseline latency value for each rat. B) Mean tail flick latencies at 30 minutes post-DLT injection were converted to % M.P.E. values. Statistical analyses of thermal latencies were performed by two-way ANOVA followed by Bonferroni *post-hoc *while statistics for transformed % M.P.E. data were accomplished by one-way ANOVA followed by Tukey's *post-hoc *multiple comparison test. Data represent means ± s.e.m. for n = 5–6 rats per group. The *asterisk *denotes a significant difference from saline-treated rats. * = p < 0.05. 0: Baseline prior to drug administration; MS: morphine sulfate; PF: propentofylline.

The acute effects of the selective δOR agonist [D-Ala] ^2^- deltorphin II (DLT; 10 μg/30 μl) on thermal nociceptive thresholds in rats receiving chronic morphine, with or without the concomitant administration of propentofylline or vehicle are depicted in Figure 2. Baseline latencies were similar in all treatment groups, indicating no effect of pretreatment on normal thermal nociception (Figure 2A). Rats that received prolonged morphine treatment had significantly higher latencies at 30 minutes post-DLT injection when compared to rats pretreated with saline (Treatment F_3,15 _= 8.739, p < 0.001; Time F_5,15 _= 19.41, p < 0.001), indicating a morphine-induced enhancement in δOR-mediated analgesia. Concomitant treatment of morphine with propentofylline significantly attenuated the morphine-induced enhancement in δOR ligand effects. Animals pretreated with morphine displayed significantly greater percent maximum possible effect (% M.P.E.) values than did saline-treated animals (F_3,20 _= 6.700, p = 0.0026; Figure 2B). The enhancement in DLT-mediated analgesia following morphine treatment was blocked when animals were co-treated with propentofylline. These animals displayed % M.P.E.s similar to control animals, indicating no change in δOR activity. Chronic administration of propentofylline alone had no effect on the analgesic effects of DLT, yielding % M.P.E.s similar to control animals.

## Discussion

Opioid agonists are highly efficacious analgesics; however their clinical use is limited by the incidence of adverse effects, particularly the development of analgesic tolerance following repeated use. A growing body of evidence identifies an important role for the δOR in modulating morphine tolerance [5-10] and this phenomenon may involve the trafficking of δORs from internal stores toward the neuronal plasma membrane, thereby enhancing the effects of δOR-selective ligands [4,14-18,33]. The mechanism by which this contributes to morphine tolerance is unknown; however recent studies support an active role for spinal glia following chronic morphine treatment [31,32]. In the current study, we investigated the relationship between δORs and glial activation and indeed demonstrate a functional role for spinal glia in morphine-induced changes in δOR agonist effects. Moreover, administration of a glial inhibitor effectively blocked these changes in δOR function.

The involvement of spinal glia in the modulation of morphine analgesia has been demonstrated in both preclinical [25,27,31,32,34-36] and clinical [37] studies. We hypothesized that the recruitment of glial cells is a gradual response to long-term morphine administration and may be detectable at time points earlier than those at which analgesic tolerance is established. We therefore assessed the spinal immune response using a 48 h morphine dosing schedule; one which has been shown to have substantive effects on δOR trafficking and function [14-16,38]. This dosing regimen does not produce a state of tolerance [15]; however it may initiate mechanisms involved early in the cascade of events leading to opioid tolerance. In developing a means of assessing the three dimensional structures of GFAP- and OX42-immunoreactive cells, we observed significant increases in cell volume and surface area of fluorescent GFAP- and OX42-immunoreactive cells in the dorsal spinal cord following prolonged morphine treatment. These results are in accordance with previous studies [31,32] illustrating the recruitment of glia in the events precipitating opioid tolerance. Morphine-induced glial hypertrophy was attenuated by co-administration with propentofylline. Interestingly, while propentofylline administration alone had no effect on astrocytes, it produced significant microglial hypertrophy in comparison with saline-treated rats. It is not clear why this occurs, since the combination of morphine and propentofylline did not show such an effect. The neuroprotective role of microglia in the CNS is well known and this cell population is very much attuned to its microenvironment, responding swiftly to even subtle physiological changes [39]. It is possible that the localized administration of an exogenous compound into the spinal canal, in the absence of any 'pathological' events, was sufficient to produce a microglial response, although such an observation has not been reported previously [31]. Nevertheless, additional functional studies are necessary to determine whether this propentofylline-induced increase in cell size was indeed accompanied by an inflammatory response. Despite microglial hypertrophy, however, neither baseline tail flick latencies nor deltorphin-mediated analgesia were altered following propentofylline administration alone, suggesting that this increase in microglial cell size was not functionally relevant in our study.

Activation of both glial cells and δORs appears to be important in the mechanisms of morphine tolerance, however it is unknown whether these two events are mutually exclusive or if, in fact, they represent important and related intermediary steps in the development of tolerance. Previous studies demonstrate that δORs are trafficked from internal stores toward the neuronal plasma membrane following morphine treatment, correlating with an increased functional competence of the receptor [14-16]; however it is not known if the spinal immune response observed following morphine is requisite for this δOR trafficking event. Therefore, our second series of experiments aimed to examine the functional role of spinal glia in morphine-induced changes in δOR function. Consistent with earlier reports [14,15,40,41], we observed a significant augmentation in δOR-mediated effects in rats treated with morphine. This enhancement was effectively blocked by co-administration of morphine with propentofylline, demonstrating an integral role of spinal glial activation in the functional changes in δOR.

Taken together with previous reports that glial inhibition prevents the development of morphine tolerance [27,31,32], this study provides additional evidence for the role of δORs in opioid tolerance and suggests that glial activity may precipitate changes in the δOR, including receptor trafficking. Glial cell activity has been documented to modulate the trafficking of ionotropic channels such as AMPA receptors [42,43]; however the current study is the first to our knowledge to suggest such a modulation of a G protein coupled receptor. The precise mechanism by which glial-modulated functional changes in δOR may occur is unclear; however two possible mechanisms include i) increased efficiency with which the receptor couples to intracellular signaling cascades, and/or ii) enhanced cell surface expression of the receptor. Future experiments will be required to investigate these possibilities.

## Conclusion

In the present study, we demonstrate a relationship between δOR function and spinal glial activation. Indeed, prolonged administration of morphine induced the activation of astrocytic and microglial cells in the lumbar spinal cord, which correlated with enhanced antinociceptive effectiveness of a δOR agonist. Moreover, attenuation of glial activation with propentofylline, a glial inhibitor, attenuated the enhancement of δOR agonist-mediated effects. These data support an intimate relationship between glial and opioidergic function and provide insight into the mechanisms by which opioid analgesic tolerance develops.

## Methods

### Animals

Adult male Sprague-Dawley rats (220–300 g; Charles River, Québec, Canada), were housed in groups of two with *ad libitum *access to food and water, and maintained on a reverse 12/12 h light/dark cycle. All behavioural experiments were performed during the dark phase of the cycle, and animals were handled prior to experimentation in order to reduce stress-related analgesia. All experimental protocols were approved by the Queen's University Animal Care Committee, and complied with the policies and directives of the Canadian Council on Animal Care and the International Association for the Study of Pain.

### Drug treatments

Rats were separated into one of four groups receiving i) morphine and intrathecal saline, ii) morphine and intrathecal propentofylline (inhibitor of glial activation), iii) intrathecal propentofylline alone, or iv) intrathecal saline alone (control group). Morphine sulfate (MS) was administered every 12 h by subcutaneous injections of increasing doses (5, 8, 10, 15 mg/kg in saline; Sabex, Kingston General Hospital, Kingston, Ontario, Canada). This treatment protocol was previously shown to induce the trafficking of δORs from intracellular compartments to neuronal plasma membranes in cultured cortical neurons as well as in the spinal cord [14]. Propentofylline and saline (10 μg/30 μl diluted in saline and 30 μl, respectively; Sigma, St. Louis, MO, USA) were administered by intrathecal injection via lumbar puncture between the L4 and L5 vertebrae under brief isofluorane anesthesia every 24 hours for 5 days based on drug administration protocols required to block morphine tolerance [31]. Successful drug placement was confirmed by a vigorous tail flick upon injection. All experiments were performed 12 hours following the final morphine injection.

### Double-labeling fluorescent immunohistochemistry for confocal microscopy

Rats (n = 3 per group) were deeply anesthesized with sodium pentobarbital (75 mg/kg, i.p.; MTC Pharmaceuticals, Cambridge, Ontario, Canada) and transaortically perfused with 4% paraformaldehyde (PFA) in 0.1 M phosphate buffer (PB; 500 ml, pH 7.4). The spinal cords were removed by spinal ejection and post-fixed in the above fixative for 30 minutes at room temperature and cryoprotected in 30% sucrose in 0.2 M PB for 48 hours at 4°C. Lumbar segments were isolated and cut into 40 μm transverse sections on a freezing sledge microtome and collected in 0.1 M Tris buffered saline (TBS; pH 7.4). Free-floating sections were incubated in a blocking solution containing 10% NGS, 10% BSA followed by incubation with a rabbit polyclonal antisera recognizing glial fibrillary acidic protein (GFAP; 1:2500 working dilution; DakoCytomation, Ontario, Canada) to label activated astrocytes and a mouse monoclonal antisera recognizing OX42 (1:1000 working dilution; Serotec, NC, USA) to label CD3/CDIIB receptors on activated microglia. Spinal cord sections were incubated overnight at 4°C with both primary antibodies, followed by incubation with goat anti-mouse and goat anti-rabbit secondary antibodies (both 1:200 working dilution; Molecular Probes, Invitrogen, Ontario, Canada) conjugated to Alexa 594 and Alexa 488 fluorophores, respectively. To assess non-specific labeling, control sections were processed in the absence of primary antibodies. Sections were mounted on glass slides, air-dried, and cover-slipped using Aquamount (Fisher Scientific, Ontario, Canada).

Immunoreactive cells were visualized in the deep dorsal horn using the Leica TCS SP2 multi photon confocal microscope (100 × magnification; Leica Microsystems Inc, Ontario, Canada) and images acquired and digitalized for quantitative analysis with Leica Confocal Software. Twenty-five to thirty-five serial images were captured in 0.75 μm increments throughout the z plane using identical acquisition parameters and x-y coordinates for each of 12–20 immunoreactive cells per rat for n = 3 rats per experimental group. The serial images were then stacked and reconstructed in three dimensions using Image-Pro Plus v5.0 software (MediaCybernetics, MD, USA). Total cell volume (in pixels^3^) and cell surface area (in pixels^2^) were calculated for each cell based on the three dimensional cell reconstruction. Statistical analyses were performed using Excel XP (Microsoft, Ontario, Canada) and Prism 4.01 (Graph Pad, San Diego, CA). The average volume and surface area for cells within each treatment group were calculated and expressed as means ± s.e.m. These values were compared by one-way ANOVA followed by Tukey's *post-hoc *multiple comparison test. P values less that 0.05 were considered significant.

### Behavioural tail flick assay

The effects of a selective δOR agonist, DLT (10 μg/30 μl [i.t.]; Sigma), on thermal nociceptive responses were assessed using the hot water tail flick assay [15]. The distal 5 cm segment of the rat's tail was immersed in noxious 52°C water, and the latency to a vigorous tail flick was measured. For n = 6 per group, three baseline latencies were measured prior to DLT injection in order to determine the normal nociceptive responses of the animals. A cut-off latency of four times the average baseline response threshold was imposed to avoid tissue damage in the event that the animal became unresponsive following DLT injection. Rats were then injected intrathecally with DLT. Thermal latencies were measured every 10 minutes following drug administration for 50 minutes. The % M.P.E. of DLT was calculated at the 30 minute time point, as this time point corresponded with the maximum analgesic effect of DLT.

% M.P.E. = (post-drug latency - baseline) ÷ (cut-off latency - baseline) × 100

The thermal latencies to respond were analyzed by two-way ANOVA followed by Bonferroni *post-hoc *and the transformed % M.P.E. data were analyzed by one-way ANOVA followed by Tukey's *post-hoc *multiple comparison test. All values are expressed as means ± s.e.m. P values less than 0.05 were considered significant.

## Abbreviations

α-amino-3-hydroxy-5-methylisoxazole-4-propionic acid (AMPA); Bovine serum albumin (BSA); central nervous system (CNS); [D-Ala]^2^-deltorphin II (DLT); dorsal root ganglia (DRG); glial fibrillary acidic protein (GFAP); intrathecal (i.t.); maximum possible effect (M.P.E.); morphine sulfate (MS); natural goat serum (NGS); opioid receptor (OR); paraformaldehyde (PFA); propentofylline (PF); subcutaneous (s.c.); Tris buffered saline (TBS).

## Competing interests

The author(s) declare that they have no competing interests.

## Authors' contributions

SVH: project conception and design; data analysis and interpretation; writing, editing, revision of manuscript

SAA: major data collection; data analysis; editing, revision of manuscript

AMWT: preliminary data collection; data analysis

CMC: project conception and design; data interpretation; editing, revision of manuscript

*All authors read and approved the final manuscript
